# Adalimumab Decorated Nanoparticles Enhance Antibody Stability and Therapeutic Outcome in Epithelial Colitis Targeting

**DOI:** 10.3390/pharmaceutics14020352

**Published:** 2022-02-02

**Authors:** Markus Ries, Brice Moulari, Maryam A. Shetab Boushehri, Mohamed Ehab Ali, Daniel Molnar, Arnaud Béduneau, Yann Pellequer, Alf Lamprecht

**Affiliations:** 1 Department of Pharmaceutics, Institute of Pharmacy, University of Bonn, 53121 Bonn, Germany; markusries@uni-bonn.de (M.R.); maryam.shetab@uni-bonn.de (M.A.S.B.); ehabali@uni-bonn.de (M.E.A.); 2 PEPITE EA4276, University of Burgundy/Franche-Comté, 25030 Besançon, France; brice.moulari@univ-fcomte.fr (B.M.); arnaud.beduneau@univ-fcomte.fr (A.B.); yann.pellequer@univ-fcomte.fr (Y.P.); 3 Boehringer Ingelheim Pharma GmbH & Co. KG, 88400 Biberach, Germany; daniel.molnar@boehringer-ingelheim.com

**Keywords:** adalimumab, inflammatory bowel disease, colitis, inflammation, surface-decorated nanoparticles

## Abstract

Inflammatory bowel disease (IBD) is a chronic inflammatory disease of the gastrointestinal tract with increasing incidence worldwide. Although a deeper understanding of the underlying mechanisms of IBD has led to new therapeutic approaches, treatment options are still limited. Severe adverse events in conventional drug therapy and poor drug targeting are the main cause of early therapy failure. Nanoparticle-based targeting approaches can selectively deliver drugs to the site of inflammation and reduce the risk of side effects by decreasing systemic availability. Here, we developed a nanoparticulate platform for the delivery of the anti-TNF-α antibody adalimumab (ADA) by covalent crosslinking to the particle surface. ADA binding to nanoparticles improved the stability of ADA against proteolytic degradation in vitro and led to a significantly better therapeutic outcome in a murine colitis model. Moreover, immobilization of ADA reduced systemic exposure, which can lead to enhanced therapeutic safety. Thus, nanoparticle protein decoration constitutes a platform through which epithelial delivery of any biological of interest to the inflamed gut and hence a local treatment can be achieved.

## 1. Introduction

Inflammatory bowel disease (IBD), in its two main forms i.e., ulcerative colitis (UC) and Crohn’s disease (CD), is a progressive, relapsing-remitting disorder of the gastrointestinal tract, characterized by epithelial and submucosal damage and intestinal inflammation [1]. As the incidence is high in industrialized countries and increasing in developing countries, UC has evolved into a global burden [2]. Although the exact etiology of UC remains unknown, multiple factors have been hypothesized to be contributors including genetics, environment and aberrant immune response [3]. Cytokines play a crucial role in the pathogenesis of UC, driving intestinal inflammation and the associated clinical symptoms [4,5]. Tumor necrosis factor (TNF)-α has been identified as a key cytokine in the treatment regimen of moderate to severe UC [6]. Conventional pharmacotherapy consists of anti-inflammatory and immunosuppressive treatment, e.g., 5-aminosalicylic acid, budesonide or azathioprine, with the goal of inducing and maintaining remission of inflammation or colonic mucosal healing [7,8]. Beyond standard therapy, the development of biologicals, including the antibody adalimumab (ADA), which specifically binds to TNF-α and neutralizes its biological function, has greatly improved treatment options in UC patients [9]. However, severe adverse events in pharmacotherapy can lead to early therapy failure with the need for surgical intervention [10,11,12]. In addition, long-term systemic immune suppressive therapy can increase the risk of infections or malignancies [13]. Adverse effects in conventional pharmacotherapy are mainly induced by unintended delivery of a given drug to non-inflamed areas of the colon. To address this problem, research is currently focusing on the development of targeted delivery systems, which can reduce adverse effects by increasing drug concentrations at the sites of inflammation and reducing systemic exposure [14].

UC is typically associated with reduced intestinal barrier integrity and abnormal mucous membrane permeability in inflamed regions of the colon [15,16,17]. In this context, nanoparticle-based targeting approaches have proven their potential to selectively deliver the drug to the site of inflammation from the epithelial side via the epithelial Enhanced Permeability and Retention (epEPR) effect [18]. The passive accumulation of nanoparticles in the inflamed tissues is directly associated with the severity of inflammation and allows the preferential uptake by immune cells, which are highly enriched in inflamed regions of the colon [19,20,21]. Increased local drug concentration at the site of action reduces systemic exposure and can dramatically improve the safety and efficacy of the therapeutic strategy [22,23,24,25,26]. 

The encapsulation of drugs inside of polymeric or lipid nanoparticles is widely considered a useful technology for targeted delivery; however, a major drawback is the high risk of premature drug release, which might cause a loss of therapeutic efficacy and provoke adverse effects. This early and unintentional drug leakage is especially increased when dealing with hydrophilic drugs given their intrinsically high solubility in aqueous luminal environment. This is further promoted by the fact that as a result of significant reduction of the size, the inner aqueous phase that is supposed to contain the hydrophilic active can be absent within the structure of the small nanoparticulate carriers [27]. First attempts to circumvent this major problem have been made by covalent immobilization of a hydrophilic drug on the surface of nanoparticles via amide bonds which prevented an early drug leakage and subsequently initiated drug release upon enzymatic linker degradation at the inflamed colonic tissue [28,29]. Beside the nanocarrier-based epithelial delivery of small molecules that suffer from the mentioned unintentional leakage to the non-inflamed tissue regions, macromolecular drugs have been of interest due to their negligible oral absorption leading to a mainly local effect, e.g., colonic delivery of low molecular weight heparins [26,30]. It would be desirable to extent this epithelial delivery approach to more specific macromolecular therapeutics, e.g., anti-inflammatory monoclonal antibodies, which are also susceptible to fast degradation in the luminal fluid of the colonic environment

Therefore, we developed polyester-based nanoparticles as a base for the superficial immobilization of the ADA and tested their therapeutic potential in an experimental murine model of acute colitis. Such nanoparticles were intended to fulfill two functions concomitantly; (i) passive targeting towards the inflamed colonic tissue via the epEPR effect and (ii) protecting the ADA immobilized on the particle surface from early degradation in the luminal content. To this end, such ADA conjugated nanoparticles (ADA-NP) were first explored in view of the influence of covalent conjugation on the activity and stability of ADA in vitro. Thereafter, ADA-NP and ADA solutions were investigated in terms of their therapeutic efficacy, their effect upon the tissue cytokine levels and their systemic absorption levels that can be responsible for potential ADA-related adverse effects.

## 2. Materials and Methods

### 2.1. Materials

Adalimumab (Cyltezo^®^, ADA) was kindly provided by Boehringer Ingelheim Pharma GmbH and Co. KG (Biberach, Germany). Poly-lactide-co-glycolide (Resomer^®^ RG 502 H, PLGA) was purchased from Evonik (Essen, Germany). 1-ethyl-3-(3-dimethylaminopropyl)-carbodiimide hydrochloride (EDC), N-α-Tosyl-L-lysine chloromethyl ketone hydrochloride (TLCK), papain and polyvinyl alcohol (Mowiol^®^ 4-88, PVA) and lipopolysaccharide (LPS) from Salmonella enterica serotype abortus equi were purchased from Sigma Aldrich (St. Louis, MO, USA). Human TNF-α ELISA was purchased from Thermo Fisher (Waltham, MA, USA). Adalimumab ELISA was purchased from AffinityImmuno (Charlottetown, Canada). Mouse TNF-α and IL-1b ELISA were purchased from Merck (Darmstadt, Germany). All other chemicals and organic solvents were of analytical grade.

### 2.2. Nanoparticle Preparation

Blank polymeric nanoparticles (BL-NP) were prepared by the oil-in-water (O/W) emulsion solvent evaporation technique [31], using poly-lactide-co-glycolide (PLGA) as a polymer. To form the organic phase, 100 mg of PLGA was dissolved in 5 mL of ethyl acetate. This organic solution was further emulsified into 10 mL of the outer water phase containing 1% *w*/*v* polyvinyl alcohol (PVA) using an ultrasonic cell disruptor (Sonopuls HD 2200, Bandelin, Berlin, Germany). The organic solvent was then evaporated under reduced pressure (Büchi Rotavapor RE 120, Büchi, Flawil, Switzerland). Subsequently, the particles were sedimented through centrifugation at 21,000× *g* for 30 min and the supernatant and excess PVA was replaced by fresh demineralized water. The amount of water added was varied to obtain BL-NP suspensions with concentrations of 10, 20 or 40 mg/mL PLGA.

### 2.3. Immobilization of Adalimumab and BSA

Adalimumab coupled nanoparticles (ADA-NP) were prepared by covalently binding of adalimumab (ADA) on the surface of BL-NP, using 1-ethyl-3-(3-dimethylaminopropyl)-carbodiimide hydrochloride (EDC) as a crosslinker. EDC utilizes the carboxylic groups on the surface of the BL-NP to react with the primary amine groups of the Lysine side chains of ADA to form stable amide bonds between the nanoparticle and the antibody. In short, 100 µL of a freshly prepared EDC-solution (1 mg/mL) was added to 600 µL of the BL-NP suspension (10, 20 and 40 mg/mL PLGA), followed by the addition of the protein solution at the respective concentrations. Samples were incubated for 1 h at room temperature, followed by a washing step to remove excess EDC and the byproducts from the NP suspension. This method was optimized in terms of incubation conditions and educt concentrations to obtain high yields and to achieve different nanoparticle surface loading rates of 25% (ADA-NP25), 50% (ADA-NP50) or 100% (ADA-NP). Bovine serum albumin (BSA) surface-decorated PLGA nanoparticles (BSA-NP) were similarly prepared. The efficiency of immobilization of protein on the nanoparticle surface and the extent of surface saturation was determined by a protein quantification assay (Roti^®^-Quant universal, Thermo Fisher, Waltham, MA, USA) according to the manufacturer’s instructions. Following crosslinking and centrifugation, the excess of soluble, unbound protein was measured in the supernatant.

### 2.4. Physicochemical Characterization of the Nanoparticles

Nanoparticle suspensions and protein solutions were analyzed for their size and size distribution by photon correlation spectroscopy (PCS) at a fixed angle of 173° at 25 °C (SZ-100, Horiba, Kyoto, Japan). The nanoparticle size was measured in terms of Z-Average, mean diameter (MD) and polydispersity index (PDI). The yield of the nanoparticle preparation (solid content) was determined gravimetrically via freeze-drying of nanoparticle suspensions (LYOVAC^®^ GT 2, Steris GmbH, Hürth, Germany).

### 2.5. Field Emission Scanning Electron Microscopy

ADA-NP suspension was pipetted onto a glass coverslip and airdried overnight. Coverslips were glued on scanning electron microscopy (SEM) aluminum stubs using Acheson silver conducting paint (Plano GmbH, Wetzlar, Germany) and sputter coated with platinum (Quorum Q150T S, Laughton, East Sussex, UK) for 20 s. Secondary electron (SE) imaging was performed with a Helios G4 Dual beam (Thermo Fisher Scientific, Eindhoven, The Netherlands) at 2.5 mm working distance and 2 kV acceleration voltage.

### 2.6. Assessment of Adalimumab In Vitro Activity

ADA and ADA-NP with different surface loading rates of 25% (ADA-NP25), 50% (ADA-NP50) or 100% (ADA-NP) were incubated with equivalent volumes of a human TNF-α solution for 1 h at 37 °C to reach equilibrium. Different molar concentrations of ADA solution and ADA-NP (log ADA −2 to 6 pM) were analyzed at a constant TNF-α concentration. The amount of soluble, unbound TNF-α was determined using a human TNF-α ELISA (Thermo Fisher, Waltham, MA, USA) according to the manufacturer’s instructions. The dose-response curves were plotted and fitted with GraphPad Prism 8 (GraphPad Software, San Diego, CA, USA) using the four-parameter equation for sigmoidal fit.

### 2.7. Stability of Adalimumab against Proteolytic Activity

The cysteine protease papain (≥10 units/mg) was used to simulate proteolytic conditions in the colonic tissue cells. ADA solution and ADA-NP (100% surface loading rate) were incubated with papain in a shaking water bath at 37 °C in phosphate-buffered saline (PBS) to study whether covalent immobilization of ADA affects its sensitivity to proteolytic degradation. To study concentration-dependent effects, ADA or ADA-NP to papain ratios of 2:1, 5:1 and 10:1 (*w*/*w*) were used. Samples were taken at defined time points and the reaction was stopped by N-α-Tosyl-L-lysine chloromethyl ketone hydrochloride (TLCK), which completely inhibits the proteolytic activity of papain at a concentration of 75 µg/mL. Antibody fragments were analyzed by high-performance size-exclusion liquid chromatography (HP-SEC), using an AdvanceBio SEC 300 Å 7.8 × 300 mm column (Agilent Technologies, Santa Clara, CA, USA) on a Waters Alliance System and a Waters 2996 Photodiode Array detector (Waters, Milford, MA, USA) set to 220 nm. Elution was performed with 20 mM phosphate buffer at pH 7 and at a flow rate of 0.35 mL/min. The HP-SEC method was modified in terms of flow rate, pH and ionic strength to obtain optimum peak separation.

### 2.8. TNF-α Neutralization Efficiency in Cell Culture Models

J774.A1 macrophage-like cell line was purchased from Sigma-Aldrich. The cells were cultured in Dulbecco’s minimum essential medium (DMEM) supplemented with 10% fetal bovine serum (FBS), 50 μg/mL streptomycin and 50 U/mL penicillin G in a 37 °C incubator with 5% CO_2_ and 95% humidified air. The cells were tested regularly for mycoplasma contamination using the MycoAlert Mycoplasma Detection Kit (Lonza, Basel, Switzerland).

To compare the efficiency of the free and nanoparticle-bound ADA, J774.A1 cells were seeded with a density of 2 × 10^5^ cells/well in 24-well culture dishes and left overnight for adherence. The cells were then incubated with 10 µg/mL LPS solution overnight to induce an inflammatory phenotype. After two washing steps, the cells were incubated with different concentrations of ADA solution or equivalent concentrations of nanoparticle-bound ADA (ADA-NP) for another 24 h. To determine the effect of ADA/ADA-NP on the total TNF-α induction, in one set of the experiments, the supernatant collected at this point was thoroughly vortexed and the concentration of the TNF-α was directly measured using ELISA. To investigate to what extent the free or nanoparticle-bound ADA can scavenge the secreted TNF-α (sTNF-α), TNF-α concentration in the supernatant in another set of experiments was measured following the removal of the aggregates through centrifugation at 21,000× *g* for 15 min.

### 2.9. Investigation of Cell–Nanoparticle Interactions

J774.A1 cells were seeded with a density of 2 × 10^5^ cells per well in 24-well culture dishes and left overnight for adherence. The cells were then incubated with 10 µg/mL LPS solution overnight to induce an inflammatory phenotype. After two washing steps, the cells were incubated at 37 °C with two different concentrations of coumarin 6-labled ADA-NP or the control BSA-NP (100 and 300, and 1000 µg/mL based on the amount of the polymeric matrix, equivalent to 10, 15 and 100 µg/mL ADA). After different incubation times, the cells were washed twice with cold phosphate buffered saline (PBS; pH = 7.4) and lysed with 200 µL of ethanol. The amount of cell associated nanoparticles was then determined based on the fluorescence of coumarin 6 recovered from the cell lysates and using a previously generated calibration curve. The data were normalized to the corresponding protein content of the cells measured through the microBCA assay (Thermo Fisher, Waltham, MA, USA).

For visual investigations, cells were seeded analogously but on coverslips (10 mm in diameter) and left overnight for adherence. Following the induction of an inflammatory phenotype through overnight incubation with 10 µg/mL LPS solution, cells were incubated with coumarin 6 loaded ADA-NP or BSA-NP for 30 min. For these investigations, the middle range concentration 300 µg/mL polymeric matrix, equal to 15 µg/mL ADA, was selected. Following incubation, the supernatant was removed, and the cells were washed twice with cold PBS (pH = 7.4). Subsequently, the cell membrane was stained with wheat germ agglutinin (WGA)-Alexafluor 647 (10 µg/mL), and the cells were fixed with 4% paraformaldehyde solution for 10 min. The nuclei were then stained with DAPI (300 nM), and the coverslips were mounted on slides using Mowiol^®^ mounting medium. The samples were examined using a confocal laser scanning microscope (CLSM; Nikon Eclips Ti, Nikon Corporation Inc., Tokyo, Japan).

### 2.10. Investigation of the Potential Immunogenicity

J774-DUAL™ cells were obtained from Invivogen (San Diego, CA, USA). The cells were cultivated in DMEM supplemented with 10% heat inactivated FBS, 50 μg/mL streptomycin and 50 U/mL penicillin G and 100 µg/mL Normocin™ and were further supplemented with the selective antibiotics (200 µg/mL Zeocin™ and 20 µg/mL Blasticidin™) every other week. The cells were grown in a 37 °C incubator with 5% CO_2_ and 95% humidified air and were tested regularly for mycoplasma contamination using the MycoAlert Mycoplasma Detection Kit (Lonza, Basel, Switzerland).

To investigate the potential immunogenicity of the free or particle-bound ADA, J774-DUAL™ cells were seeded in 96-well culture dishes with a density of 10^5^ cells/well. Following the induction of an inflammatory phenotype through overnight incubation with 10 µg/mL LPS solution, the cells were incubated with different concentrations of ADA or ADA-NP for 24 h. Induction of the nuclear factor kappa B (NF-κB) levels was determined through the measurement of the induced secreted embryonic alkaline phosphatase (SEAP) levels in the supernatant using the Quanti-Blue^TM^ reagent (Invivogen, San Diego, CA, USA).

### 2.11. Animals and In Vivo Study Design

All animal experiments were carried out in accordance with the recommendations of the Guide for the Care and Use of Laboratory Animals (Institute of Laboratory Animal Resources, National Research Council, National Academy of Sciences, Washington, DC, USA). Experiments were conducted at the University of Franche-Comté in Besançon, France in compliance with the French legislation on animal experimentation under the experimentation authorization no. A-25-48. An overview of the animal study design is shown in Figure S1. Colitis was induced by 2,4,6-trinitrobenzene sulfonic acid (TNBS) as previously described to generate a stable and reproducible inflammation in the murine colon [32]. Male BALB/c mice (average weight 25 g) were catheterized 4 cm intrarectally after light narcotizing with ether. 100 µL of TNBS in ethanol (1:1), enough to cover the entire colon, were injected in a dose of 90 mg/kg body weight. The mice were housed for a day without treatment for the colitis model to fully develop. Colitis-bearing mice (*n* = 6/group) were treated once a day for three consecutive days with different ADA formulations, administered either rectally (p.r.) or subcutaneously (s.c.). ADA nanoparticle formulations were given at a dose of 20 mg/kg body weight while the ADA solution was administered at doses of 2 or 20 mg/kg body weight. The colitis control groups received either saline (CTRL+) or BSA-NP and a healthy control group (CTRL−) was also included in the setup. The animals were sacrificed 24 h after the last administration and the colons were resected.

### 2.12. Assessment of Therapeutic Efficacy

The extent of inflammation and therapeutic efficacy was quantified for 5 days throughout the study using a clinical activity score. The score was calculated in terms of weight loss, stool consistency and rectal bleeding [33]. After treatment, colons were resected, opened longitudinally and rinsed with iced phosphate buffer to remove residual luminal content. The wet weight and length of each colon was measured and expressed as the colon weight/length ratio. Levels of pro-inflammatory cytokines (TNF-α and IL-1b) in the colonic tissue were analyzed using commercially available ELISA (Merck, Darmstadt, Germany) according to the manufacturer’s instructions. The myeloperoxidase (MPO) activity was determined in colon tissue as reported previously [34]. MPO activity is a well-established biomarker of ulcerative colitis and measure of neutrophil infiltration [35].

To assess the systemic exposure and to collect preliminary pharmacokinetic data, colitis mice (*n* = 3/group) were treated with either ADA-NP (rectally) or ADA solution (s.c. or p.r.) for 3 consecutive days. Blood samples were taken 3 and 12 h after the final treatment and analyzed using ADA ELISA (AffinityImmuno, Charlottetown, Canada) according to the manufacturer’s instructions.

### 2.13. Statistical Analysis

All results are expressed as mean values ± standard deviation (SD). Unpaired Student’s t test was used for direct comparisons and ANOVA followed by Tukey’s multiple comparisons test was applied to compare multiple experimental groups. GraphPad Prism 8 (GraphPad Software, San Diego, CA, USA) was used to calculate the *p*-values. Different levels of significance have been determined: *p* < 0.05, *p* < 0.01 or *p* < 0.001 with the respective symbols *, # and $.

## 3. Results

### 3.1. Particle Characterization

**Figure 1:**
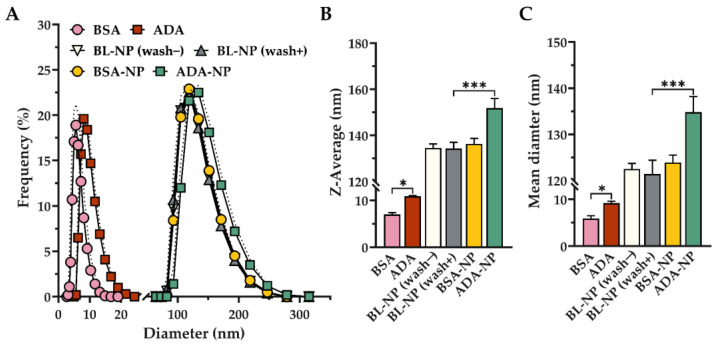
Physicochemical nanoparticle properties analyzed by PCS. (**A**) Particle size distribution curve of bovine serum albumin (BSA) or adalimumab (ADA) solution and BSA- or ADA conjugated nanoparticles (BSA-NP, ADA-NP). Data are shown as mean (solid lines) ± SD (dotted lines) for *n* = 10 measurements. (**B**) Z-Average and (**C**) Mean diameter of BSA- or ADA-solution and BSA- or ADA-NP. Data are shown as mean ± SD (*n* = 10). Statistical significance was assessed by one-way ANOVA followed by Tukey’s multiple comparisons test (* *p* < 0.05, *** *p* < 0.001).

To investigate physicochemical particle properties, BL-NP, ADA-NP and ADA solution were analyzed using photon correlation spectroscopy (PCS). In addition, BSA solution and BSA-NP were analyzed, which served as pharmacologically inactive controls. PCS measurements revealed relatively low PDI of 0.1 ± 0.1 or 0.2 ± 0.1 and narrow size distributions for all samples (Figure 1A, Table S1, Supplementary Materials). For BL-NP, a Z-Average of 134 ± 3 nm and a mean diameter of 121 ± 3 nm was determined after washing. Covalent attachment of ADA or BSA to the surface of BL-NP led to a measurable increase of the hydrodynamic diameter. A significant increase of 18 nm in Z-Average for ADA-NP and of 2 nm for BSA-NP was found relative to the BL-NP. The mean diameter significantly increased by 13 nm for ADA-NP and by 3 nm for BSA-NP. Washing steps during the preparation of BL-NP had no noticeable influence on the particle size distribution, Z-Average or mean diameter (Figure 1A,B). Solid content (yield of blank nanoparticle preparation) was 93.2 ± 5.0% (*n* = 6). Field emission scanning electron microscopy revealed spherical shaped nanoparticles with a narrow size distribution (Figure S2).

### 3.2. Adalimumab Immobilization

**Figure 2:**
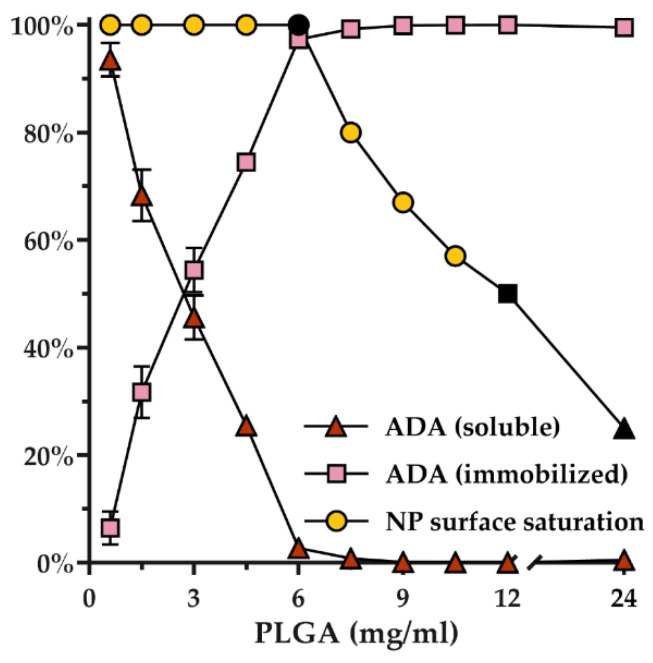
Coupling efficiency of adalimumab (ADA) on blank nanoparticles (NP) using EDC as a crosslinker. Different PLGA concentrations resulted in nanoparticles with surface saturation rates of 100% (●, ADA-NP), 50% (■, ADA-NP50) and 25% (▲, ADA-NP25). Values are calculated by quantifying the excess of soluble ADA in supernatant after centrifugation at 21,000× *g* for 30 min following EDC crosslinking. Data are shown as mean ± SD (*n* = 3).

To assess the efficiency of ADA immobilization on the nanoparticle surface, the number of soluble antibodies was quantified in the supernatant after coupling with EDC. We found that the quantity of immobilized ADA was strongly correlated with the concentration and the total surface area of the nanoparticles (Figure 2, Table S2). Complete surface saturation with high yields of 97.3 ± 0.4% was found at a PLGA to ADA ratio of 12:1, indicating that sufficient total nanoparticle surface was available to quantitatively bind the amount of antibody added. A PLGA to ADA ratio of 24:1 or 48:1 led to a nanoparticle surface saturation of approximately 50% or 25%, respectively.

### 3.3. In Vitro Activity of Adalimumab

**Figure 3:**
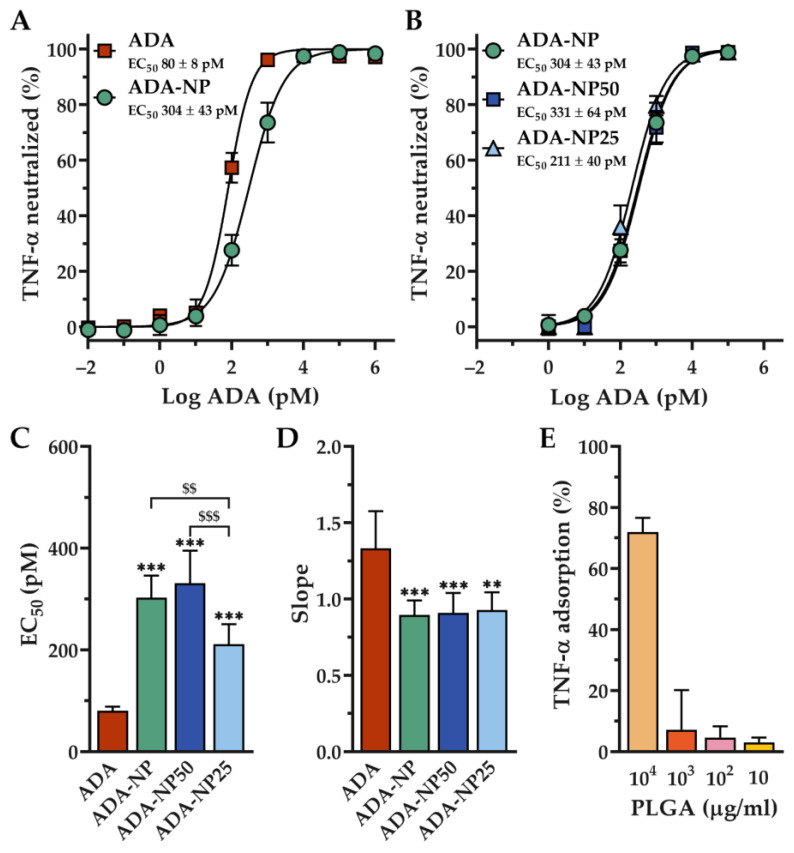
Tumor necrosis factor α (TNF-α) of adalimumab (ADA) formulations following incubation with human TNF-α and measured with ELISA. Dose–response curves of (**A**) ADA solution and ADA coupled nanoparticles with 100% surface loading rate (ADA-NP) or (**B**) ADA coupled nanoparticles with 100% (ADA-NP), 50% (ADA-NP50) and 25% surface loading rates (ADA-NP25). The dose–response curves were plotted and fitted with GraphPad Prism 8 using the four-parameter equation for sigmoidal fit. Data are shown as mean ± SD (*n* = 6). (**C**) Half maximal effective concentration (EC_50_) and (**D**) neutralization slope values for all tested ADA formulations. Data are shown as mean ± SD (*n* = 6). Statistical significance was assessed by one-way ANOVA followed by Tukey’s multiple comparisons test (** *p* < 0.01, *** *p* < 0.001 vs. ADA solution; $$ *p* < 0.01, $$$ *p* < 0.001). (**E**) Effect of passive adsorption on blank PLGA nanoparticles (BL-NP) surface at different PLGA concentrations. Data are shown as mean ± SD (*n* = 3).

Functional integrity and in vitro activity tests revealed that the extent of TNF-α neutralization was concentration-dependent for all ADA formulations as shown in sigmoidal shaped dose–response curves (Figure 3A,B). The neutralization dose-response curves were found to shift towards higher ADA concentrations when ADA was coupled to nanoparticles. We found a significant reduction of TNF-α neutralization for all ADA coupled nanoparticles at log ADA concentrations of 2 and 3 pM. In addition, higher EC_50_ (half maximal effective concentration) and lower slope values were found with all ADA coupled nanoparticles, indicating a significant loss of activity compared to free ADA in solution. The EC_50_ and slope values for each ADA formulation assayed against human TNF-α are shown in Figure 3C,D and Table S3. ADA coupled nanoparticles with loading rates of 25% (ADA-NP25) showed a decrease of the EC_50_ value compared to nanoparticles with 100% (ADA-NP) or 50% (ADA-NP50) surface loading rates. In addition, the slope value of the dose-response curve of ADA-NP25 was slightly higher compared to ADA-NP or ADA-NP50; however, this difference was not statistically significant. Interestingly, no noticeable difference in EC_50_ or slope values were found for nanoparticles with loading rates of 50% (ADA-NP50) compared to ADA-NP.

To assess the extent of nonspecific interactions between TNF-α and the nanoparticle surface, we performed a control experiment without ADA. To this end, TNF-α was incubated with blank PLGA nanoparticles (BL-NP), according to the aforementioned method. With PLGA concentrations of 100 µg/mL, only 4.6 ± 3.6% of TNF-α was adsorbed (Figure 3E). Since ADA-NP (5 pM) only contain 16.8 µg/mL PLGA, it can be assumed that the extent of unspecific binding of TNF-α to nanoparticles is negligible.

Collectively, we found 26.5% (ADA-NP) of the TNF-α-scavenging activity of ADA to be preserved after immobilization on the nanoparticle surface. Furthermore, lower antibody loading rates could increase TNF-α neutralizing potency, whereby ADA-NP25 showed significantly lower EC_50_ values compared to ADA-NP or ADA-NP50.

### 3.4. Stability of Adalimumab against Proteolytic Degradation

**Figure 4:**
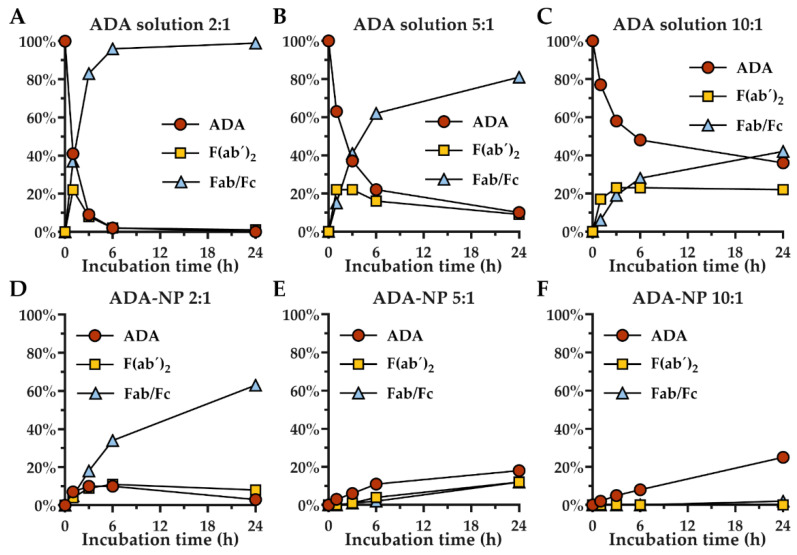
Impact of covalent immobilization of adalimumab (ADA) on its stability upon exposure to papain. (**A**–**C**) Free ADA in solution (ADA) and (**D**–**F**) ADA coupled nanoparticles with 100% surface loading rate (ADA-NP) were incubated with papain at 2:1, 5:1 or 10:1 (*w*/*w*) ratios. Digestions were conducted in PBS at 37 °C, with the inhibitor N-α-Tosyl-L-lysine chloromethyl ketone hydrochloride (TLCK) added to abrogate papain activity. Samples of proteolysis mixtures were analyzed by HP-SEC at different time intervals. Fab, fragment antigen-binding; Fc, fragment crystallizable.

Next, we studied whether covalent immobilization of ADA on nanoparticles affects its sensitivity to proteolytic degradation. The rate of papain digestion was strongly dependent on the ADA to papain ratio and led to an accumulation of F(ab´)_2_ and Fc/Fab fragments (Figure 4, Table S4). After 6 h incubation of ADA solution with papain in a 2:1 (ADA to papain) ratio, 98% of ADA was already fragmented. ADA to papain ratios of 5:1 or 10:1 resulted in delayed fragmentation, with 90% or 64% of ADA fragmented after 24 h, respectively. Interestingly, conjugation of ADA to the surface of nanoparticles strongly enhanced the stability against proteolytic degradation. Only 45% fragmentation was observed after 6 h incubation of ADA coupled nanoparticles with 100% surface loading rate (ADA-NP) with papain in a 2:1 ratio. ADA-NP to papain ratios of 5:1 and 10:1 resulted in an even slower degradation. After 24 h incubation of ADA-NP with papain at a 10:1 ratio, only 2% of ADA was fragmented. Here, covalent immobilization of ADA on nanoparticles reduced proteolytic degradation 30-fold compared to free ADA in solution. Unexpectedly, after incubation of ADA-NP with papain, intact ADA as well as ADA fragments were detected in the solution.

Overall, these results confirm that immobilization of ADA on the surface of NP can significantly enhance the resistance of the antibody against proteolytic degradation.

### 3.5. TNF-α Neutralization in Cell Models

**Figure 5:**
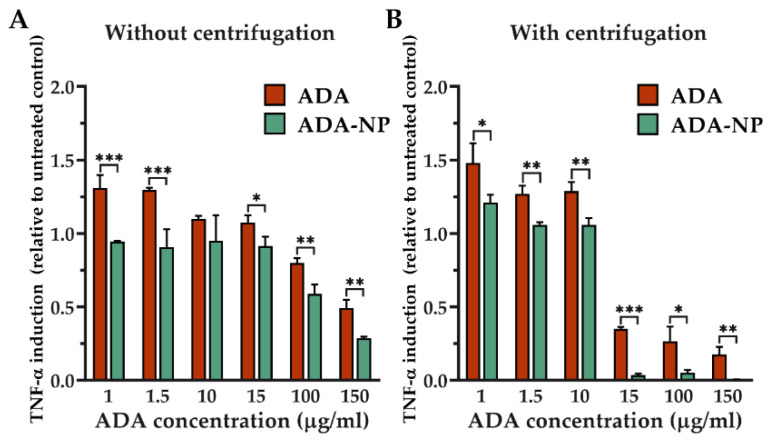
Tumor necrosis factor α (TNF-α) levels in the supernatant of J774.A1 macrophages pretreated with 10 µg/mL LPS and incubated overnight with different concentrations of free or particle-bound ADA (**A**) without or (**B**) with 15-min-long centrifugation at 21,000× *g*. The results have been expressed as fold relative to the untreated control. Data are shown as mean ± SD (*n* = 3). Statistical significance was assessed by unpaired *t*-test (* *p* < 0.05, ** *p* < 0.01, *** *p* < 0.001). ADA, adalimumab; ADA-NP, ADA conjugated nanoparticles; LPS, lipopolysaccharide.

Figure 5 summarizes the ability of the free and particle-bound ADA to suppress/neutralize the TNF-α secreted by the inflammatory J774.A1 macrophages. As observed in Figure 5, the concentration of the TNF-α in the supernatant of the cells that have been treated with ADA-NP was significantly lower than that obtained from the macrophages having been incubated with corresponding concentrations of the ADA solution. To investigate how much of this is related to the better scavenging ability of the nanoparticle-bound ADA, the TNF-α concentration was also measured in the obtained supernatants following centrifugation at 21,000× *g* for 15 min to remove the aggregates in the samples obtained from both treatment groups. The results demonstrated that the scavenging effect was in fact most pronounced at high ADA concentrations (>15 µg/mL) and that the nanoparticle-bound ADA had significantly better TNF-α scavenging and neutralization efficiency than the free ADA. In addition to the direct scavenging and neutralization of the sTNF-α, the particle-bound ADA seemed to have a better general ability to reduce the TNF-α secretion from the inflammatory J774.A1 macrophages at high concentrations (>15 µg/mL), as evident from the significantly lower concentration of the TNF-α in the uncentrifuged well-vortexed supernatants. In this case, though both ADA and ADA-NP seemed to reduce the TNF-α secretion at higher concentrations, the reduction was significantly more pronounced in case the ADA is bound to the nanoparticle surface.

### 3.6. Cell–Nanoparticle Interactions

**Figure 6:**
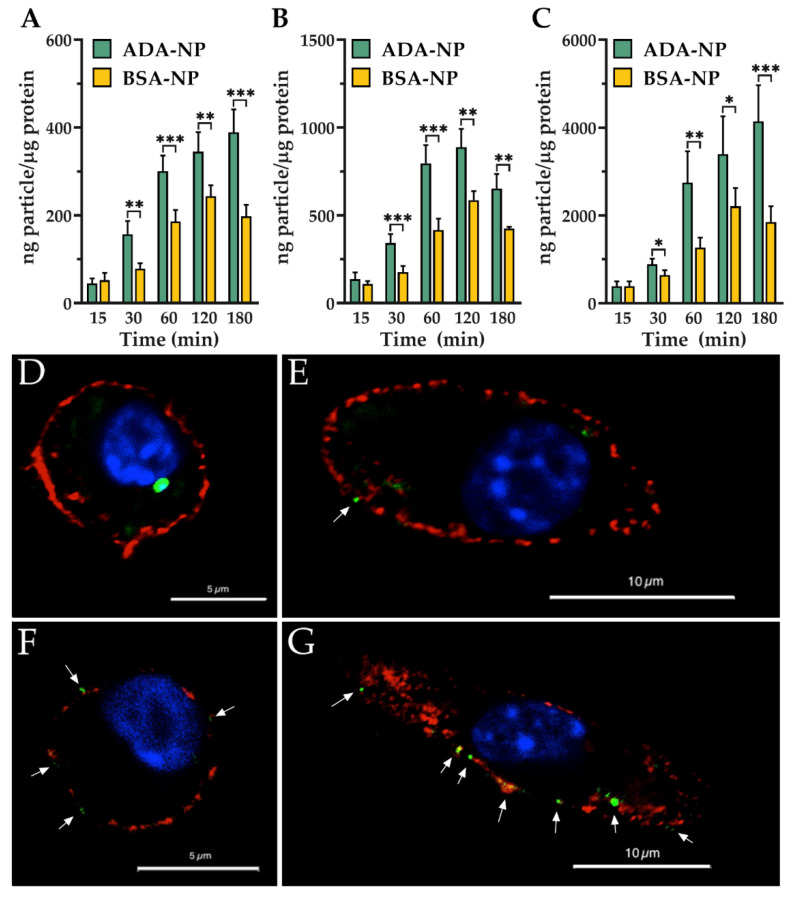
(**A**–**C**) Combined uptake and binding of adalimumab (ADA) conjugated nanoparticles (ADA-NP) to the inflammatory macrophages in comparison with the control bovine serum albumin (BSA) conjugated nanoparticles (BSA-NP): Cells were treated with 100 µg/mL (**A**), 300 µg/mL (**B**) or 1000 µg/mL (**C**) coumarin 6-loaded ADA-NP or BSA-NP at 37 °C for different time intervals ranging from 15 to 180 min. ADA-NP showed significantly higher cellular association at all time points (starting from 30 min) when compared to the control BSA-NP. Statistical significance was assessed by unpaired T-test (* *p* < 0.05, ** *p* < 0.01, *** *p* < 0.001). (**D**–**G**) CLSM images comparing the interaction of the ADA-NP (**F**,**G**) and the control BSA-NP (**D**,**E**) with inflammatory macrophages: Imaging was carried out on fixed cells following 30 min of incubation with 300 µg/mL nanoparticles (based on the polymeric matrix) at 37 °C. The blue color represents the cell nuclei, the red color the cell membrane and the green color the coumarin 6-loaded nanoparticles. Qualitatively seen, ADA-NP seemed to have significantly higher membrane binding compared to the control BSA-NP.

Figure 6A-C depicts the combined uptake and binding of ADA-NP in comparison with the control BSA-NP over a period of 180 min for three different highest nanoparticle concentration of 100 (Figure 6A), 300 (Figure 6B) and 1000 µg/mL (Figure 6C; based on the concentration of the polymeric matrix, equivalent to 8.3, 25 and 83.3 µg/mL ADA). The concentration of 3000 µg/mL nanoparticle matrix was not investigated due to its toxicity for the cells. As observed, ADA-NP had significantly higher association with the J774.A1 macrophages when compared to the BSA-NP. To explore as to whether this might be related to the higher membrane binding of the ADA-NP, CLSM imaging was used to enable a comparison of the nanoparticle localization 30 min post-incubation with the inflammatory macrophages. As observed in Figure 6D–G, ADA-NP had higher membrane association when compared to the control BSA-NP.

### 3.7. Potential Immunogenicity of ADA and ADA-NP

The potential immunogenicity of the free and particle-bound ADA was investigated through the measurement of the NF-κB induction levels in the inflammatory J774.DUAL^TM^ cells (Figure S3). As observed, neither the free nor the particle-bound ADA resulted in a significant induction of NF-κB, which demonstrates their minimal immunogenicity at least in the macrophage cell line used for the cell culture experiments.

### 3.8. In Vivo Therapeutic Efficacy

**Figure 7:**
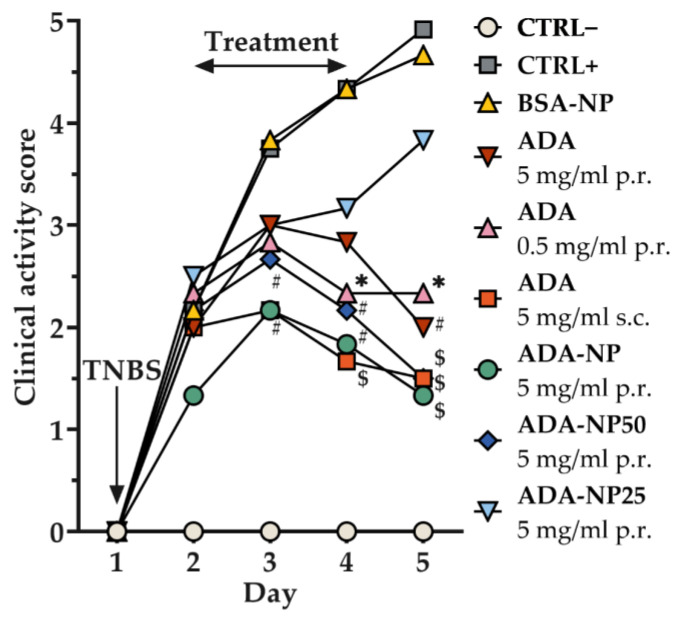
Therapeutic efficacy of adalimumab (ADA) treatment on clinical signs of colitis expressed as the clinical activity score, a composite measure of weight loss, stool consistency and rectal bleeding. Colitis was induced by rectal administration of TNBS (90 mg/kg body weight) and the mice were treated for 3 consecutive days. Data are shown as mean ± SD (*n* = 6). Error bars are not shown for clarity reasons. Statistical significance was assessed by one-way ANOVA followed by Tukey’s multiple comparisons test (* *p* < 0.05, # *p* < 0.01, $ *p* < 0.001, vs. CTRL+). ADA-NP, ADA conjugated nanoparticles with 100% surface loading rate; ADA-NP50, ADA conjugated nanoparticles with 50% surface loading rate; ADA-NP25, ADA conjugated nanoparticles with 25% surface loading rate; BSA, bovine serum albumin; BSA-NP, BSA conjugated nanoparticles; CTRL−, healthy control, CTRL+, colitis control.

**Figure 8:**
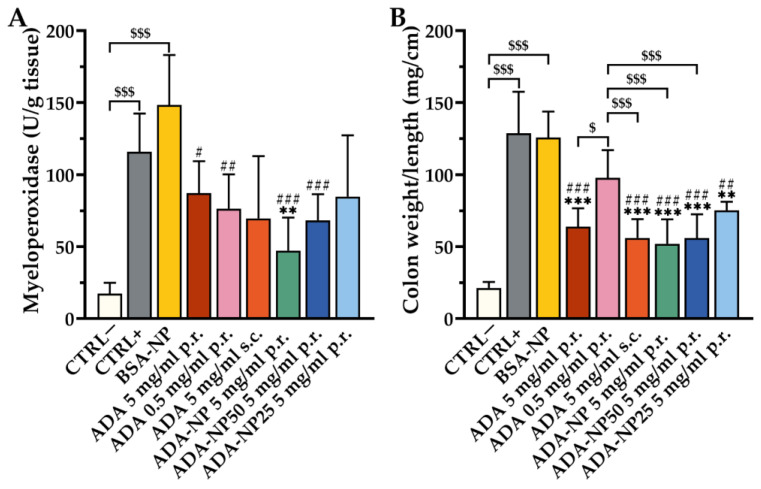
Anti-inflammatory effect of adalimumab (ADA) treatment on myeloperoxidase (MPO) activity and the colon weight/length ratio. Colitis was induced by rectal administration of TNBS (90 mg/kg body weight), and the mice were treated for 3 consecutive days. (**A**) MPO activity in colon tissue cells measured with MPO activity assay. (**B**) The colon weight/length ratio is represented as milligrams per centimeter of colon. Data are shown as mean ± SD (*n* = 6). Statistical significance was assessed by one-way ANOVA followed by Tukey’s multiple comparisons test (** *p* < 0.01, *** *p* < 0.001 vs. CTRL+; # *p* < 0.05, ## *p* < 0.01, ### *p* < 0.001 vs. BSA-NP; $ *p* < 0.05, $$$ *p* < 0.001). ADA-NP, ADA conjugated nanoparticles with 100% surface loading rate; ADA-NP50, ADA conjugated nanoparticles with 50% surface loading rate; ADA-NP25, ADA conjugated nanoparticles with 25% surface loading rate; BSA, bovine serum albumin; BSA-NP, BSA conjugated nanoparticles; CTRL−, healthy control, CTRL+, colitis control.

**Figure 9:**
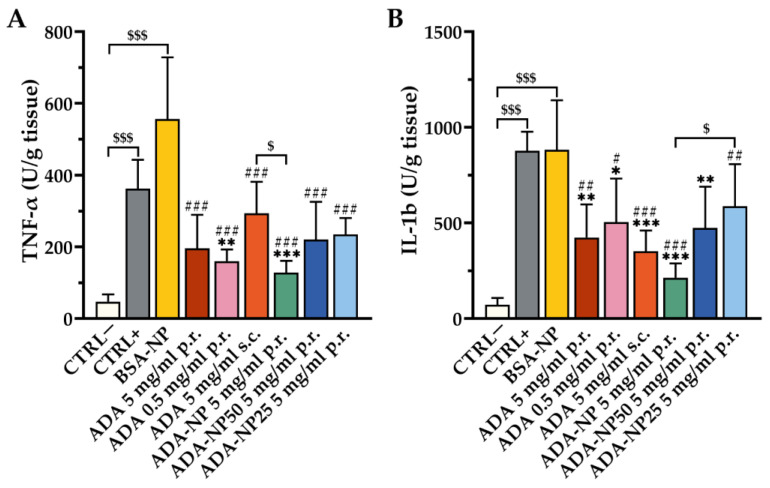
Anti-inflammatory effect of adalimumab (ADA) treatment on cytokine levels. Intestinal concentrations of (**A**) tumor necrosis factor α (TNF-α) and (**B**) interleukin-1b (IL-1b) in colon tissue of colitis mice. Colitis was induced by rectal administration of TNBS (90 mg/kg body weight), and the mice were treated for 3 consecutive days. Cytokines were measured in colon tissue using ELISA. Data are shown as mean ± SD (*n* = 6). Statistical significance was assessed by one-way ANOVA followed by Tukey’s multiple comparisons test (* *p* < 0.05, ** *p* < 0.01, *** *p* < 0.001 vs. CTRL+; # *p* < 0.05, ## *p* < 0.01, ### *p* < 0.001 vs. BSA-NP; $ *p* < 0.05, $$$ *p* < 0.001). ADA-NP, ADA conjugated nanoparticles with 100% surface loading rate; ADA-NP50, ADA conjugated nanoparticles with 50% surface loading rate; ADA-NP25, ADA conjugated nanoparticles with 25% surface loading rate; BSA, bovine serum albumin; BSA-NP, BSA conjugated nanoparticles; CTRL−, healthy control, CTRL+, colitis control.

The validated TNBS-colitis mouse model was applied to investigate the therapeutic efficacy of ADA-based treatment with disease-specific endpoints. TNBS-treated mice developed a moderate to severe colitis, as evidenced by elevated levels of the clinical activity score (Figure 7), the colon weight/length ratio, myeloperoxidase (MPO) activity (Figure 8) and pro-inflammatory cytokines (Figure 9). As control, either saline solution (CTRL+) or BSA-NP were administered. The extent of inflammation was consistent in both control groups with no significant difference between BSA-NP and CTRL+. ADA formulations were given rectally or subcutaneously (s.c.) on three consecutive days to study the effect of the route of administration on the therapeutic outcome. All ADA formulations except ADA-NP25 led to a significant reduction of the clinical activity score compared to the control groups. The most significant and fastest response to treatment was observed after rectal administration of ADA coupled nanoparticles with 100% surface loading rate (ADA-NP) with increasing treatment efficacy over time. MPO activity was strongly reduced by all ADA formulations, though only treatment with ADA-NP showed a significant effect compared to both control groups. All ADA formulations except ADA solution (p.r., 0.5 mg/mL) significantly reduced the colon weight/length ratio by 1.5- to 2-fold compared to saline or BSA-NP control groups. Treatment with ADA-NP and ADA-NP50 led to the strongest reduction of the colon weight/length ratio, which was superior to treatment with ADA solution (p.r., 0.5 mg/mL). Levels of the pro-inflammatory cytokines, TNF-α and interleukin-1b (IL-1b) in the colonic tissue were significantly elevated in both colitis control groups (CTRL+, BSA-NP) compared to untreated control (CTRL–). ADA treatment led to a significant decrease in the levels of TNF-α and IL-1b compared to CTRL+ or BSA-NP. The most significant effect on the reduction of cytokine levels was observed after treatment with ADA-NP, and this effect was superior to the other treatments.

Collectively, these findings demonstrate that rectal administration of ADA alleviates predominant clinical symptoms of experimental colitis in the mouse model and suppresses inflammation at the cellular level, with ADA-NP showing the highest therapeutic efficacy.

### 3.9. Assessment of Systemic Adalimumab Exposure

**Figure 10:**
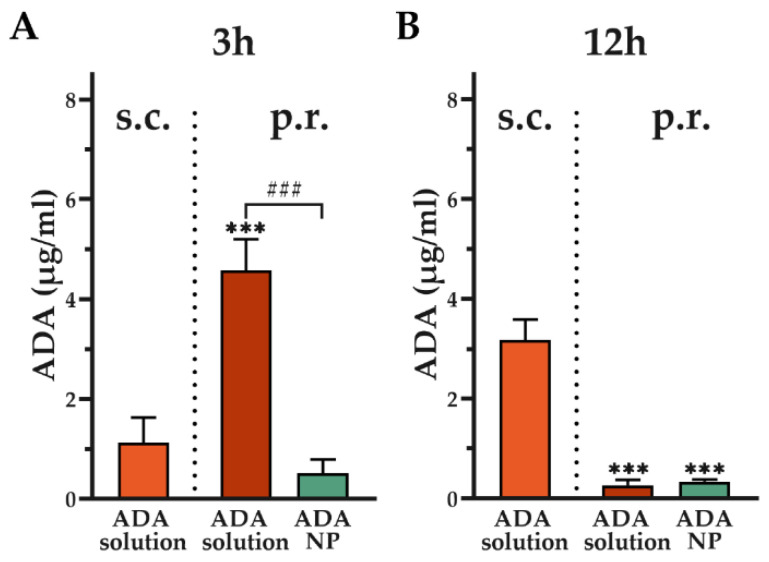
Adalimumab (ADA) concentration in serum from colitis mice (**A**) 3 and (**B**) 12 h after final administration. Colitis was induced by rectal administration of TNBS (90 mg/kg body weight) and the mice were treated with ADA coupled nanoparticles with 100% surface loading rate (ADA-NP, p.r.) or ADA solution (ADA, s.c. or p.r.) for 3 consecutive days. Blood samples were taken 3 and 12 h following last administration and ADA concentrations were measured using ELISA. Data are shown as mean ± SD (*n* = 3). Statistical significance was assessed by one-way ANOVA followed by Tukey’s multiple comparisons test (*** *p* < 0.001 vs. ADA solution s.c.; ### *p* < 0.001).

To assess systemic ADA exposure, colitis mice (*n* = 3) were treated for three days with ADA solution and ADA coupled nanoparticles with 100% surface loading rate (ADA-NP) and blood samples were collected 3 and 12 h after the final treatment. Rectal (p.r.) administration of ADA solution resulted in faster uptake compared to subcutaneous (s.c.) administration (Figure 10A) of ADA solution. Three hours after the last rectal administration of ADA solution, systemic ADA levels were significantly increased by more than 4-fold compared to s.c. administration of ADA solution. Nanoparticle-binding of ADA resulted in a significant reduction in absorption rate when administered rectally, with ADA levels measured three hours after the last rectal administration being 9-fold lower compared to ADA solution. 12 h after the final treatment, ADA serum levels were significantly elevated by more than 9-fold following s.c. administration of ADA solution compared to rectal administration of ADA solution or ADA-NP (Figure 10B).

Collectively, these data reveal that nanoparticle-binding significantly reduces systemic levels of ADA.

## 4. Discussion

In this study, we designed nanoparticles for the delivery of the anti-TNF-α antibody adalimumab (ADA) to target inflamed colonic epithelium and promote local anti-inflammatory immune responses in a murine colitis model. EDC crosslinking chemistry was used to covalently immobilize ADA to the surface of blank PLGA nanoparticles via amide bonds [36,37]. PLGA was chosen as polymer for the preparation of nanoparticles due to its biocompatible and biodegradable properties which does not lead to toxicologically relevant residues in the inflamed tissues when accumulated and for offering carboxylic groups for activation by EDC [38]. ADA conjugation with PLGA nanoparticles resulted in a significant but only slight increase of the hydrodynamic diameter, indicating that none or a negligible number of aggregates were formed. This increase in diameter could be considered equivalent to the diameter of a monolayer of antibodies around the NP [39,40]. After immobilization of ADA, its potency to scavenge TNF-α was significantly reduced by 73.5% (ADA-NP) compared to free ADA in solution. This is potentially the result of a random orientation of the antibody on the nanoparticle surface, which may impair the accessibility of antigen binding sites due to steric hindrance [41,42,43,44]. On the contrary, lowering the antibody densities to only 25% (ADA-NP25) increased the TNF-α neutralization potency in vitro, as indicated by significantly lower EC_50_ values for ADA-NP25. This correlation between the loss of neutralization potency and the density of ADA on the nanoparticles’ surfaces is in line with the assumption that antigen binding sites should be more accessible at lower antibody surface densities [45,46].

Accumulating evidence reveals that during inflammation, the activity of proteases is strongly upregulated, which can lead to epithelial damage and increased intestinal permeability and may contribute to the pathogenesis of UC [47]. Based on this background, we investigated proteolytic degradation of free ADA and surface-immobilized ADA in vitro, using the cysteine protease papain as a model protease. Interestingly, ADA binding to the surface of nanoparticles not only delayed the fragmentation but it also led to a cleavage of intact antibodies from the nanoparticle surface. Consistently, after rectal treatment of colitis mice with nanoparticle-bound ADA, measurable levels of free ADA were found in the systemic circulation.

Several mechanisms have been proposed to explain the therapeutic effects of TNF-α inhibitors in UC [9]. Beside neutralization of sTNF-α, interactions with transmembrane TNF-α (tmTNF-α) are crucial for the treatment of UC [48]. The results of the cell culture experiments demonstrated that both free and particle-bound ADA could lead to a significant reduction of the TNF-α secreted by inflammatory macrophages when used at high concentrations (>15 µg/mL). This general reduction was more pronounced at high ADA concentrations and was significantly higher for the particle-bound ADA compared to its free counterpart. Furthermore, centrifugation of the obtained supernatants to remove the aggregates confirmed the ability of both free ADA and ADA-NP to neutralize the already secreted TNF-α particularly at higher concentrations (>15 µg/mL). Yet again in this case, the particle-bound ADA performed superior to its free counterparts. Two main conclusions can be drawn from these findings: (i) both the ADA and ADA-NP have the ability to scavenge sTNF-α at adequate concentrations, though given the large surface area to volume ratio provided by the core nanoparticles upon which the ADA has been immobilized, the scavenging ability of the ADA-NP seems to be superior; (ii) as the ADA and ADA-NP result in a general reduction of the total sTNF-α above certain concentrations, they seem to also interact with the tmTNF-α, thereby inhibiting its shedding as sTNF-α. Indeed, it has been demonstrated that the binding of anti-TNF-α antibodies to the tmTNF-α in antigen presenting cells leads to a rapid internalization of the complex into the endosomes and subsequently the lysosomes, where it is most probably degraded [49]. Here again, the large surface area to volume ratio of the immobilizing nanoparticles might have facilitated the interaction of the decorated ADA with the tmTNF-α. In fact, investigation of the cellular interaction of the ADA-NP with the inflammatory macrophages confirmed their significantly higher association with the cells when compared to the control BSA decorated nanoparticles overtime. Additionally, CLSM imaging during the initial minutes of incubation revealed a significantly higher binding of the ADA-NP to the cell membrane compared to the BSA-NP. Collectively, these findings imply that the superior TNF-α neutralizing ability of the particle-bound ADA might be beyond the enhanced sTNF-α scavenging ability of the nanoparticulate system and also in part pertain to the higher interaction of the ADA-NP with the tmTNF-α, preventing thereby its shedding from the cell membrane. Furthermore, proteolytic cleavage of intact ADA from the nanoparticle surface may also increase the local ADA concentrations in inflamed colonic regions in vivo. Free ADA could then be potentially distributed in the inflamed colonic tissue and contribute to the therapeutic efficacy through interaction with tmTNF-α. The veracity of this hypothesis, however, needs to be further investigated.

Interestingly, immobilization of ADA on nanoparticles had a strong stabilizing effect against proteolytic degradation. In fact, previous studies have shown that immobilization can be used to stabilize proteins [50,51]; however, stabilizing effects against proteolytic degradation by antibody immobilization have been investigated poorly. Improved stability against proteolytic degradation could be caused by impaired accessibility of the papain cleavage sites due to the dense packaging of ADA on the nanoparticle surface. This too seems to be a major advantage over the potential use of PEGylated antibodies with the same goal, since the steric hindrance of proteolytic degradation would be much more limited with a polyethylene glycol chain compared to the surface area of a larger nanoparticle.

IBD is associated with increased serum and tissue levels of pro-inflammatory cytokines, including TNF-α and IL-1b, which drive intestinal inflammation and lead to an impaired epithelial barrier function [52,53,54]. This contributes to an increased intestinal permeability and enables a size-dependent accumulation of nanoparticles in inflamed regions [20]. The epithelial EPR effect has been identified as a key factor for improved therapeutic efficacy of nanoparticulate formulations in experimental colitis [18,20,55]. Similar targeting approaches have been applied to other chronic inflammatory diseases, e.g., rheumatoid arthritis, to selectively deliver drugs to sites of inflammation [56]. However, the physiological and pathophysiological context which strongly depends on the disease and tissues, is quite different in IBD, which involves here additional challenges such as the presence of microflora and high enzyme activity in the gut lumen.

Our study shows that intra-colonic administration of ADA-NP attenuated the severity of clinical symptoms of experimental colitis in mice more efficiently than ADA solution by decreasing the clinical activity score and the colon-weight/length ratio. Furthermore, ADA-NP treatment suppressed pro-inflammatory mediators including TNF-α, MPO and IL-1b more efficiently compared to ADA solution. Of all nanoparticulate formulations, ADA coupled nanoparticles with 100% surface loading rate (ADA-NP) showed the strongest therapeutic efficacy. According to the impaired accessibility of the papain cleavage sites, enhanced stability against proteolytic degradation due to dense packaging of the antibody could contribute to the superior effect of ADA-NP.

Anti-TNF-α treatment has a wide-ranging impact on the immune system and can cause severe systemic side effects including malignancies or opportunistic infections [57]. High drug levels are associated with an increased risk of adverse events; however, sufficient doses of ADA should be administered to neutralize surplus TNF-α levels and to achieve clinical remission in UC [58]. Besides, high drug levels and frequent administration of anti-TNF-α agents can lead to increased immunogenicity and loss of response (LOR) [59]. LOR is a major concern in IBD treatment and is caused by the formation of antibodies against TNF-α antagonists, resulting in rapid clearance and altered drug efficacy [60]. Therefore, optimal drug concentrations are difficult to achieve and patients on anti-TNF-α therapy must be observed closely [61]. In addition, the significant interindividual variability in the pharmacokinetic profiles of anti-TNF agents complicates therapeutic drug monitoring (TDM) [62].

A targeted nanoparticle-mediated delivery to the inflamed tissues could improve therapeutic safety by reducing systemic exposure levels. Here, we show that intra-colonic administration of ADA-NP leads to a significant reduction in systemic availability when compared to subcutaneous or rectal administration of ADA solution. Moreover, nanoparticle-binding prevents ADA from entering the systemic circulation and thus leads to enhanced local effects. In addition to a potentially improved safety profile, nanoparticle-binding of ADA could help reduce immunogenicity due to the formation of anti-ADA antibodies. Altered pharmacokinetics may also lead to decreased interindividual variability in drug pharmacokinetic profiles. Several efforts have been made towards developing nanotherapeutics for the treatment of IBD, focusing mainly on the encapsulation of the drug in nanoparticles as this method ensures a protection of the cargo [63]. We could show that nanoparticle-binding of ADA could protect the antibody from its early degradation in the luminal content and increase its stability. Furthermore, the immobilization on the surface of nanoparticles avoids the risk of premature drug release, an effect often observed with drug encapsulation in nanoparticles [64,65] leading to a subsequent increase of adverse effects after systemic availability of the drug. Moreover, in opposition to the potential use of PEGylated antibodies, the steric protective effect of the nanoparticle-backbone leads to significantly ameliorated ADA stability in the luminal content with ADA-NP.

## 5. Conclusions

This study shows that ADA-NP successfully provides several therapeutic benefits compared to a local antibody therapy in the intestine. Therapeutic efficacy is significantly improved by nanoparticle-mediated delivery of ADA to the site of action in a sustained way. Nanoparticle-binding of ADA efficiently reduces early proteolytic degradation of the active maintaining the activity of the immobilized ADA. Besides, the nanoparticle that is attached to ADA also hinders its passing through the permeabilized epithelial barrier, further enhancing the local effect of ADA as well as reducing adverse effects based on its systemic availability.

Consequently, surface-immobilization of biologicals on nanoparticles can be a useful tool for targeted drug delivery to inflamed colonic tissue, while providing protection of the cargo.

## Data Availability

Not applicable.

## Supplementary Materials

The following are available online at www.mdpi.com/article/10.3390/pharmaceutics14020352/s1, Figure S1. Animal study design. Colitis was induced by 2,4,6-trinitrobenzene sulfonic acid (TNBS) in a dose of 90 mg/kg body weight. Mice were housed for a day for the colitis model to fully develop. Animals were treated on day 2–4 and sacrificed 24 h after the last administration. Colons were then resected and analyzed in terms of weight/length, levels of pro-inflammatory cytokines and MPO. BSA, bovine serum albumin; ADA, adalimumab, p.r., per rectum, s.c., subcutaneously; Table S1: Physicochemical nanoparticle properties analyzed by PCS. Data are shown as mean ± SD (n = 10); Figure S2: Field emission scanning electron microscopy picture of adalimumab coupled nanoparticles with 100% surface loading rate (ADA-NP); Table S2: Coupling efficiency of adalimumab (ADA) on blank PLGA nanoparticles for different polymer to adalimumab ratios (A ADA-NP25, B ADA-NP50, C ADA-NP). Data are shown as mean ± SD (n = 3); Table S3: EC50 and neutralization slope values of the neutralization dose-response curves of adalimumab solution (ADA) and adalimumab coupled nanoparticles with 100% (ADA-NP), 50% (ADA-NP50) and 25% (ADA-NP25) surface loading rates assayed against human TNF-α. Data are shown as mean ± SD (n = 6); Table S4: Effect of immobilization of adalimumab (ADA) on its stability upon exposure to papain. ADA solution (ADA) and ADA coupled nanoparticles with 100% surface loading rate (ADA-NP) were incubated with papain at different ratios (*w*/*w*); Figure S3: Potential immunogenicity of the free or particle-bound ADA (ADA-NP) determined through the measurement of NF-κB induction in J774.DUALTM cells pretreated with 10 µg/mL LPS: NF-κB induction was determined through the measurement of secreted embryonic alkaline phosphatase levels in the supernatant following overnight incubation with different concentrations of ADA or ADA-NP using the Quanti-BlueTM assay. No significant increase of NF-κB levels was detected compared to the untreated control.
