# Supplementary material for: Adalimumab Decorated Nanoparticles Enhance Antibody Stability and Therapeutic Outcome in Epithelial Colitis Targeting
Source: Pharmaceutics. 2022 Feb 2;14(2):352. doi: 10.3390/pharmaceutics14020352 (PMC8879121; doi:10.3390/pharmaceutics14020352)
Supplement: Supplementary file 1 [file pharmaceutics-14-00352-s001.zip › pharmaceutics-1568651-supplementary.pdf]

pharmaceutics

Supplementary Material: Adalimumab Decorated Nanoparticles Enhance Antibody Stability and Therapeutic Outcome in Epithelial Colitis Targeting

Markus Ries, Brice Moulari, Maryam A. Shetab Boushehri, Mohamed Ehab Ali, Daniel Molnar, Arnaud Béduneau, Yann Pellequer and Alf Lamprecht

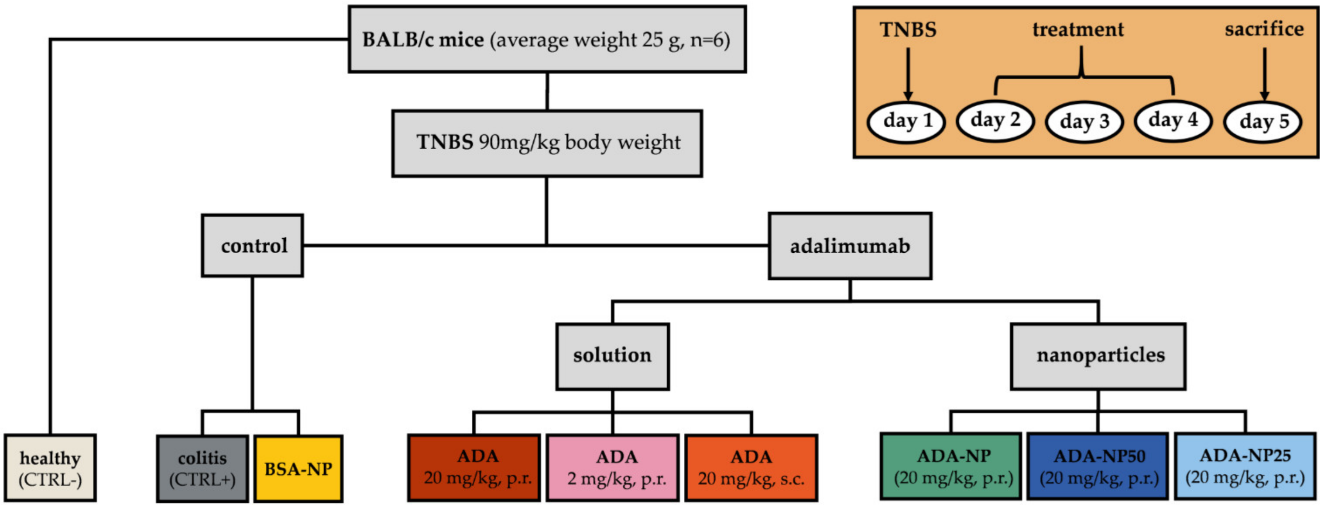

Figure S1. Animal study design. Colitis was induced by 2,4,6-trinitrobenzene sulfonic acid (TNBS) in a dose of 90 mg/kg body weight. Mice were housed for a day for the colitis model to fully develop. Animals were treated on day 2-4 and sacrificed 24h after the last administration. Colons were then resected and analyzed in terms of weight/length, levels of pro-inflammatory cytokines and MPO. BSA, bovine serum albumin; ADA, adalimumab, p.r., per rectum, s.c., subcutaneously.

Table S1. Physicochemical nanoparticle properties analyzed by PCS. Data are shown as mean ± SD (n = 10).

	BSA	ADA	BL-NP (wash−)	BL-NP (wash+)	BSA-NP	ADA-NP
**Z-Average (nm)**	7 ± 0	11 ± 0 $	135 ± 2	134 ± 3	136 ± 2	152 ± 4 ***
**Mean diameter (nm)**	6 ± 1	9 ± 0 $	123 ± 1	121 ± 3	124 ± 2	135 ± 3 ***
**Polydispersity index**	0.2 ± 0.1	0.1 ± 0.0	0.1 ± 0.1	0.1 ± 0.1	0.1 ± 0.0	0.2 ± 0.1

$ *p<0.05* vs. BSA solution; *** *p<0.001* vs. BL-NP (wash+).

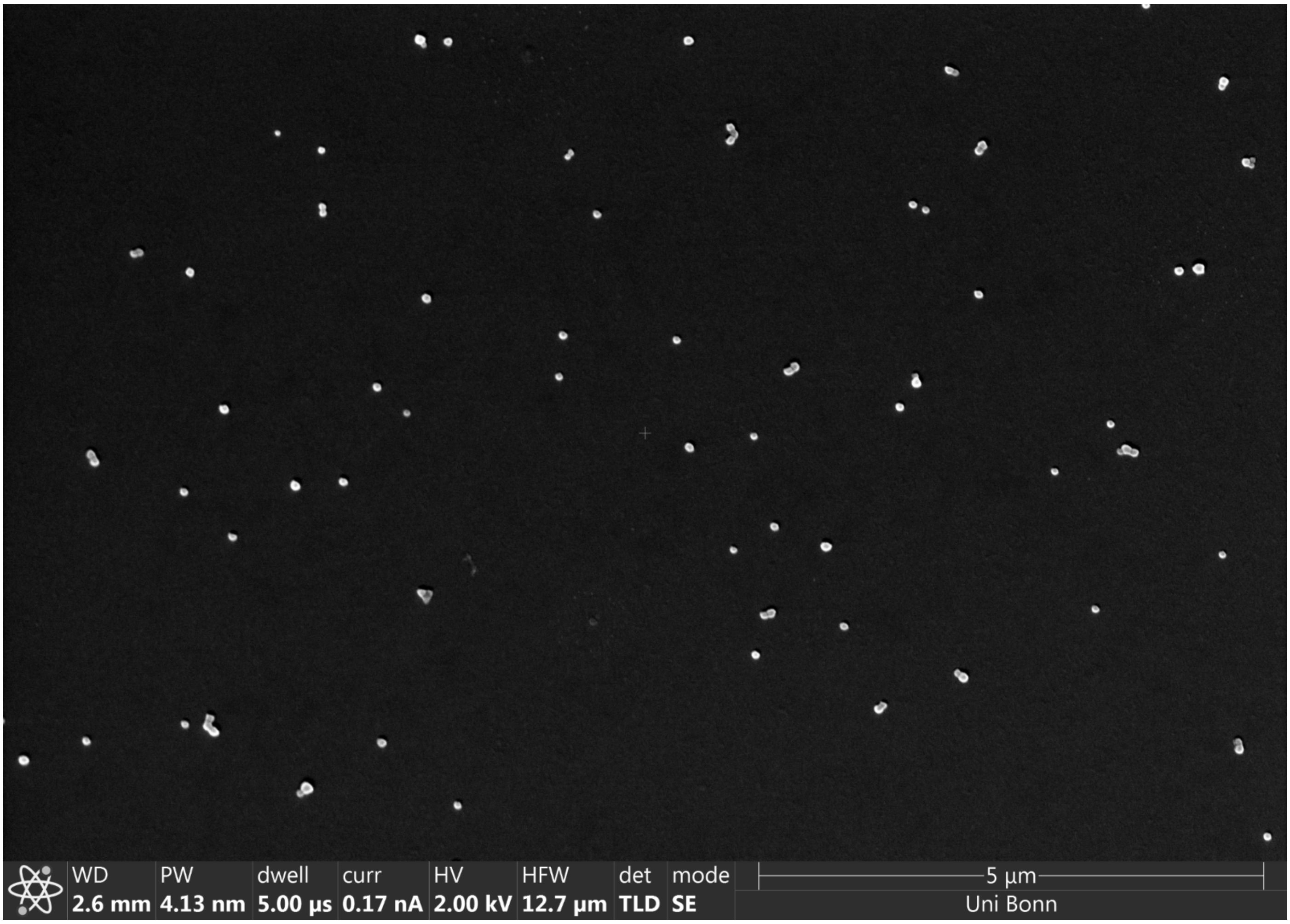

Figure S2. Field emission scanning electron microscopy image of adalimumab coupled nanoparticles with 100% surface loading rate (ADA-NP).

Table S2. Coupling efficiency of adalimumab (ADA) on blank PLGA nanoparticles for different polymer to adalimumab ratios .

PLGA (mg/ml)	Ratio (PLGA:ADA)	Percent Nanoparticle Loading (w/w)	Immobilized ADA (%)	NP Surface Saturation (%)
24.0[A]	48:1	2.1	99.5 ± 0.1	25
12.0[B]	24:1	4.2	100.3 ± 0.2	50
10.5	21:1	4.8	99.9 ± 0.2	57
9.0	18:1	5.6	99.9 ± 0.1	66
7.5	15:1	6.7	99.2 ± 0.4	80
6.0[C]	12:1	8.3	97.3 ± 0.4	100
4.5	9:1	8.3	74.5 ± 0.2	100
3.0	6:1	8.3	54.4 ± 4.1	100
1.5	3:1	8.3	31.7 ± 4.8	100
0.6	1.2:1	8.3	6.5 ± 3.1	100

([A] ADA-NP25, [B] ADA-NP50, [C] ADA-NP). Data are shown as mean ± SD (n = 3).

Table S3. EC_{50} and neutralization slope values of the neutralization dose-response curves of adalimumab solution (ADA) and adalimumab coupled nanoparticles with 100% (ADA-NP), 50% (ADA-NP50) and 25% (ADA-NP25) surface loading rates assayed against human TNF-α. Data are shown as mean ± SD (n = 6).

Sample	EC_{50} (pM)	Relative EC_{50} vs. ADA [%]	Slope	R^2
ADA	80.2 ± 8.0	100	1.33 ± 0.24	0.99
ADA-NP	302.8 ± 43.1 ***	26.5	0.90 ± 0.10 ***	0.99
ADA-NP50	331.2 ± 63.9 ***	24.2	0.91 ± 0.13 ***	0.99
ADA-NP25	210.9 ± 39.5 ***	38.0	0.93 ± 0.12 **	0.99

**$p<0.01$, ***$p<0.001$ vs. ADA solution.

Table S4. Effect of immobilization of adalimumab (ADA) on its stability upon exposure to papain. ADA solution (ADA) and ADA coupled nanoparticles with 100% surface loading rate (ADA-NP) were incubated with papain at different ratios (w/w).

		ADA solution			ADA NP		
	Incubation time (h)	ADA [%]	F(ab´)2 [%]	Fab/Fc [%]	ADA [%]	F(ab´)2 [%]	Fab/Fc [%]
ADA:papain ratio 2:1	0	100	0	0	0	0	0
	1	41	22	37	7	4	5
	3	9	8	83	10	9	18
	6	2	2	96	10	11	34
	24	0	1	99	3	8	63
ADA:papain ratio 5:1	0	100	0	0	0	0	0
	1	63	22	15	3	0	0
	3	37	22	41	6	1	1
	6	22	16	62	11	4	2
	24	10	9	81	18	12	12
ADA:papain ratio 10:1	0	100	0	0	0	0	0
	1	77	17	6	2	0	0
	3	58	23	19	5	0	0
	6	48	23	28	8	0	0
	24	36	22	42	25	0	2

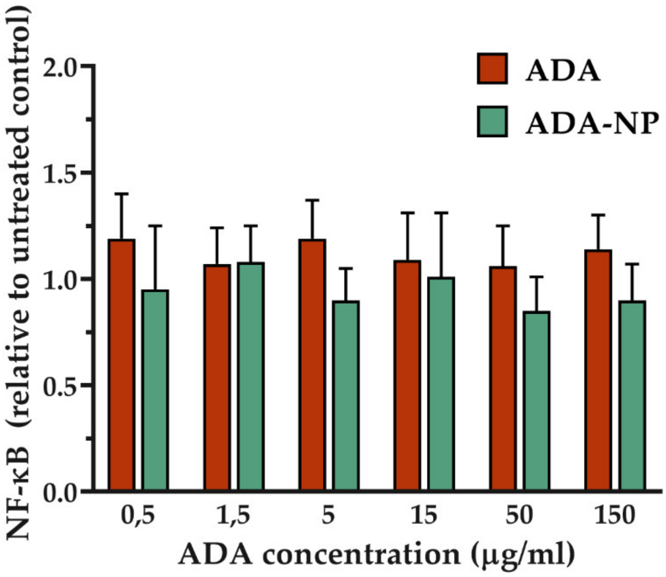

Figure S3. Potential immunogenicity of the free adalimumab (ADA) or particle-bound ADA (ADA-NP) determined through the measurement of NF-κB induction in J774.DUAL™ cells pretreated with 10 µg/ml LPS: NF-κB induction was determined through the measurement of secreted embryonic alkaline phosphatase levels in the supernatant following overnight incubation with different concentrations of ADA or ADA-NP using the Quanti-Blue™ assay. No significant increase of NF-κB levels was detected compared to the untreated control.
